# Serum lncRNA-ANRIL and SOX9 expression levels in glioma patients and their relationship with poor prognosis

**DOI:** 10.1186/s12957-021-02392-2

**Published:** 2021-09-23

**Authors:** Youlu Sun, Yuesong Jing, Yuxin Zhang

**Affiliations:** 1 Department of Neurosurgery, Guangrao County People’s Hospital, No. 180 Huayuan Road, Dongying, Guangrao County 257300 P.R. China; 2 Department of Neurosurgery, The Second People’s Hospital Of Dongying, Dongying, 257335 P.R. China

**Keywords:** lncRNA-ANRIL, SOX9, Glioma, Prognosis, Proliferation, Invasion

## Abstract

**Background:**

lncRNA-CDKN2B antisense RNA 1 (ANRIL) and SRY-box transcription factor 9 (SOX9) has abnormal expression in many tumors including glioma, but the underlying molecular mechanism is unclear. This study set out to investigate the serum lncRNA-ANRIL and SOX9 levels in glioma patients and their effects on prognosis.

**Methods:**

We enrolled 142 glioma patients admitted to our hospital from May 2014 to May 2016 into the research group (RG) and 120 healthy subjects receiving concurrent physical examinations into the control group (CG). Fasting peripheral blood (4 mL each) was sampled from subjects from the two groups. Using the quantitative real-time polymerase chain reaction (qRT-PCR), lncRNA-ANRIL and SOX9 were measured to explore their values in the early diagnosis of glioma. Patients from RG were followed up for 3 years to analyze the influence of lncRNA-ANRIL and SOX9 on patient prognosis. We purchased glioma cell lines U251 and U87 and grouped them according to the transfection of different plasmids. We conducted CCK8 assay to test cell proliferation, Transwell assay to test cell invasion, the flow cytometry to test cell apoptosis, and Western Blot assay to measure bcl-2 and bax protein levels.

**Results:**

ANRIL and SOX9 were evidently higher in RG than in CG (*P*<0.01). The receiver operating characteristic (ROC) curve revealed that the diagnostic sensitivity of ANRIL combined with SOX9 for glioma was 81.62%, and the specificity was 90.83% (*P*<0.01). ANRIL and SOX9 were closely related to tumor grade, tumor diameter, distant metastasis, and family history of glioma (*P*<0.01). In total, 135 patients were successfully followed up (95.07%). Patients with high levels of ANRIL and SOX9 had a markedly poorer prognosis than those with low levels (*P*<0.05). ANRIL and SOX9 were markedly higher in glioma cell lines (U251 and U87) than in normal brain cells (*P*<0.01). The proliferation and invasion of U251 cells were notably reduced after the transfection of ANRIL and SOX9 inhibitory sequences (*P*<0.01), but the apoptosis was notably increased (*P*<0.01). Bcl-2 expression was markedly increased in lncRNA-ANRIL-inhibitor and SOX9-inhibitor (*P*<0.01), while bax expression was markedly reduced in lncRNA-ANRIL-inhibitor and SOX9-inhibitor (*P*<0.01).

**Conclusion:**

lncRNA-ANRIL and SOX9 levels were higher in glioma patients than in healthy people. High-lncRNA-ANRIL and SOX9 levels were strongly associated with unfavorable prognosis of patients. The testing of biological behaviors revealed that lncRNA-ANRIL and SOX9 worked as tumor-promoting genes in glioma.

## Introduction

Glioma, mainly arising from neural stem or progenitor cells [1], is the most common malignancy of the central nervous system [2, 3] and accounts for about 80% of intracranial malignancies. It is featured with high morbidity, high recurrence rate, and high mortality, and its relative 5-year survival is as low as 5%. Glioma mostly attacks adults, especially those aged 30–40 years [4, 5]. Most gliomas are featured with an infiltrative spreading to the surrounding central nervous parenchyma [6]. The World Health Organization (WHO) divides glioma into 4 grades: grades I and II are low-grade gliomas, including astrocytomas and oligodendroglioma, with a long course of disease, and grades III and IV are high-grade gliomas, including anaplastic gliomas and glioblastomas [7]. Untreated gliomas can easily lead to deaths [8]. Existing clinical treatments of gliomas mainly include surgery combined with radiotherapy and chemotherapy [9, 10]. However, complete removal of glioma through surgery is difficult due to its invasive growth, not alone the high recurrence rate after the surgery [11, 12]. The exploration of the mechanisms of the proliferation and invasion of glioma cells has been a hot topic recently, aiming to identify genes and molecular mass targets that inhibit glioma tumor growth.

Long non-coding RNA (lncRNA) is involved in regulating various biological processes of the body [13, 14]. The abnormal expression of lncRNAs plays a crucial part in the progression of a variety of cancers including gliomas [15, 16]. LncRNA-CDKN2B antisense RNA 1 (ANRIL) is located in the inhibitors of cyclin dependent kinase 4. The study by Dong et al. [17] proposed that the antisense lncRNA in lncRNA-ANRIL variants is associated with gliomas. SRY-box transcription factor (SOX) genes, belonging to a family of transcription factors that contain HMG (high-mobility group) domains, have many target molecules and play a role in cell differentiation during embryonic development [18, 19]. SOX9 has abnormal expression in many tumors including glioma [20], but the underlying molecular mechanism is unclear [21].

In the process of tumor pathogenesis, the multiplication and invasion of tumor cells are crucial, which determines whether the tumor cell cycle can progress smoothly. Here we first tested the expression of lncRNA-ANRIL and SOX9 in normal adults and glioma patients to analyze the effects of the two genes on the prognosis of glioma patients. After transfecting glioma cell lines with ANRIL and SOX9 inhibitory sequences, we measured apoptosis-related proteins (bcl-2 and bax) to observe changes in multiplication, invasion, and apoptosis in glioma cell lines. We aimed to enlighten new ways to regulate glioma cell proliferation and invasion, to explore the possibility of lncRNA-ANRIL and SOX9 to act as new targets for glioma gene therapy, and to provide a reliable reference for future clinical research and diagnosis of gliomas.

## Materials and methods

### Basic information

We enrolled 142 glioma patients admitted to our hospital from May 2014 to May 2016 into the research group (RG) and 120 healthy subjects receiving concurrent physical examinations into the control group (CG). The average age of all research subjects was (34.58±5.65) years. This experiment has been approved by the hospital ethics committee, and the signed informed consent was obtained from all subjects or their immediate family members.

### Inclusion and exclusion criteria

Inclusion criteria: All patients were treated in our hospital for the first time and were all in line with the diagnosis of glioma after pathological examination. No benign or malignant tumor disease was found in this group of healthy subjects. Exclusion criteria: (1) Patients with prior treatments such as surgery; (2) patients with comorbid cardiovascular, liver, kidney, and other diseases; (3) patients with incomplete pathological data; (4) patients with other malignancies with brain metastases; (5) patients with systemic infection or nervous system disease; (6) patients with mental disorders who cannot cooperate with treatment; and (7) patients with end-stage disease.

### Experimental reagents and materials

Normal human astrocytes (HA) and U251 and U87 of glioma cell lines were provided by BeNa Culture Collection (Beijing, BNCC337972, BNCC100123, BNCC337885). DMEM and PBS were obtained from Gibco (USA, 1142802). Trypsin was from Shanghai Shifeng Biological Technology Co., Ltd. (EB04590). RPMI1640 culture medium, ANNEXIN V-FITC/PI apoptosis detection kit, and BCA protein kit were purchased from Shanghai Jingke Chemical Technology Co., Ltd. (GNM-31800, AD10-2, JK-201a). Trizol kit and reverse-transcription kit were manufactured by Shanghai Kanglang Biotechnology Co., Ltd. (KL058, KL266). LncRNA quantitative real-time polymerase chain reaction (qPCR) kit was from Beijing Bai’ao Laibo Technology Co., Ltd. (WH0125-MXG). Cell counting kit-8 (CKK8) was from Goyoo Bio Co., Ltd. (GY025). ECL Ultra Reagent was manufactured by Shanghai Chuan Qiu Biotechnology Co., Ltd. (HE-60). The HRP-labeled secondary antibody was from Shanghai Yuduo Biotechnology Co., Ltd. (YDJ3235). The microplate reader was from BioTek Instruments, Inc. (USA, PerkinElmer). FC500MCL flow cytometer was from BD (USA, FACS Canto II). Design, and synthesis of all primer sequences were undertaken by Sangon Biotech (Shanghai).

### Cell culture and transfection

#### Cell culture

Glioma cells (U251 and U87) were cultivated in DMEM comprising 10% PBS in an incubator at 37°C with 5% CO_2_.

#### Cell transfection

The day before transfection, glioma cells were plated at 3×10^4^–5×10^4^ cells/well (50% confluent) in 24-well plates, with 100 μL of culture media each well. We transfected glioma cells with lncRNA-ANRIL-inhibitor, lncRNA negative control (lncRNA-NC), SOX9-inhibitor, and SOX9 negative control (SOX9-NC), respectively, and incubated them for 24 h.

### Detection method

#### qRT-PCR determination of lncRNA-ANRIL and SOX9 expression levels

Measurements of lncRNA-ANRIL and SOX9 levels in the serum and cells were done by qRT-PCR. Collect 5 mL of peripheral blood, and extract total RNA from the peripheral blood using the instructions of Trizol reagent. UV-3100PC (UV-Vis spectrophotometer) was utilized for the determination of RNA concentration and purity. The cDNA was then generated from the RNA via reverse-transcription, and then the qRT-PCR reaction was performed using lncRNA fluorescence quantitative detection kit (SYBR Green). GAPDH was used as a reference gene and cDNA as a template for PCR reaction. The reaction system was comprised of 10 μL of SYBRPrimix Ex Taq (2×), 0.4 μL of 5′ primer, 0.4 μL of 3′ primer, and 2.0 μL of DNA template. Primer sequences are presented in Table 1. PCR conditions: 95°C for 30 s, followed by 40 cycles of 95°C for 5 s, and 60°C for 40 s. Each reaction was repeated 3 times, and the expression level was computed by the 2^−ΔΔCT^ method.

**Table 1:** Primer sequences

	Forward primer	Reverse primer
LncRNA-ANRIL	5′-TGCTCTATCCGCCAATCAGG-3′	5′-GGGCCTCAGTGGCACATACC-3′
SOX9	5′-AGTACCCGCACTTGCACAAC-3′	5′-CGTTCTTCACCGACTTCCTC-3′
GAPDH	5′-GAGTCAACGGATTTGGTCGT-3′	5′-TTGATTTTGGAGGGATCTCG-3′

#### CCK8 assay for cell proliferation

The cells were harvested 24 h after the transfection and seeded in 96-well plates at 4×10^6^ cells/well. At four timepoints (0, 24, 48, and 72 h after the cell culture), 10 μL of CCK8 solution and 90 μL of basal medium (DMEM) were added to each well, and the plates were cultured at 37°C for 4 h. Then the optical density (OD) of cells in each group was measured by a microplate reader at 450 nm.

#### Transwell assay for cell invasion

Twenty-four hours after the transfection, cells were planted in 24-well plates at 3×10^4^ cells/well. Then cells were digested by 0.25% trypsin and added to the apical chamber, together with 200 μL of RPMI1640 solution, while 500 mL of RPMI1640 solution containing 10% PBS was placed into the basolateral chamber. At 37°C, the whole system was then cultured with 5% CO_2_ for 24 h. The matrix and cells in the apical chamber that did not migrate through the membrane surface were wiped away, followed by 3 times of PBS washing. A 10-min fixation was performed using paraformaldehyde, followed by 3 washes by double distilled water. The system was stained with 0.5% crystal violet when it got dried, then the cell invasion was observed under a microscope.

#### Flow cytometry for cell apoptosis

The treated cells were subjected to digestion with 0.25% trypsin and 2 washes with PBS. Next, 100 μL of binding buffer was added to prepare a suspension of 1×10^6^ cells/mL. This is followed by the addition of Annexin V-FITC and PI to perform the incubation at room temperature in the dark. Finally, the FC500MCL flow cytometry detection was repeated 3 times to obtain the mean value.

#### Western Blot (WB) assay to detect bcl-2 and bax levels

The RIPA lysis was performed on cells in each group to extract the total protein, whose concentration was then determined by BCA method. The protein concentration was made to 4 μg/μL to perform the electrophoretic separation by 12% SDS-PAGE. After the electrophoresis, the protein was transferred to a PVDF membrane and stained by the Ponceau S. Then the protein was immersed in PBST for 5 min and washed, and blocked with 5% skim milk powder at ambient temperature for 2 h. After that, the system was blocked with bcl-2 (1:500), bax (1:500), and GAPDH primary antibody (1:1000) at 4°C overnight. Thereafter, the primary antibody was washed and the horseradish peroxidase-labeled secondary antibody (1:5000) was added to perform the incubation at 37°C for 1 h, followed by 3 times of PBS rinse, 5 min each. The extra liquid on the film was blotted with a filter paper, and the ECL was conducted to perform development in the dark. The protein bands were finally scanned to analyze the relative expression of bcl-2 and bax, with GAPDH as the internal reference.

### Endpoints

SOX9 and lncRNA-ANRIL in the peripheral blood of the two cohorts of subjects was observed. Patients in IG were followed up for 3 years. Patients were divided into high- and low-lncRNA-ANRIL groups by the median expression of lncRNA-ANRIL, or into high- and low-SOX9 groups by the median expression of SOX9, for the comparison of the 3-year survival of patients. The relationship between lncRNA-ANRIL and SOX9 and clinical pathology in IG patients was analyzed. Cell proliferation, invasion, and apoptosis after transfection were observed. Bcl-2 and bax levels were measured.

### Statistical processing

Statistical analysis was done by SPSS24.0 software (Shanghai Yuchuang Network Technology Co., Ltd.) and the data visualization by GraphPad 5 software. Intra-group comparisons were made by the independent *t* test, multi-group comparisons by one-way ANOVA (denoted by F), and pairwise comparisons by the LSD *t* test. Comparisons between multiple time points were performed by the repeated measures analysis of variance, and the post-hoc test by the Bonferroni test. The receiver operating characteristic (ROC) curve was drawn to demonstrate the diagnostic value of lncRNA-ANRIL and SOX9 for glioma. Differences were statistically significant when *P* values <0.05.

## Results

### 1. Comparison of clinical data

The two cohorts of subjects were comparable since they were not significantly different in age, sex ratio, tumor grade, tumor diameter, tumor location, distant metastasis, family history of glioma, smoking, and drinking (*P*>0.05) Table 2.

**Table 2:** Comparison of clinical data [*n*(%)]

	Research group (*n*=142)	Control group (*n*=120)	*t* or *χ*^2^	*P*
Age (year)	36.29±5.33	35.12±4.62	1.881	0.061
Sex			0.728	0.393
Male	89 (62.68)	69 (57.50)		
Female	53 (37.32)	51 (42.50)		
Tumor grade				
Grade I to II	79 (55.63)			
Grade III to IV	63 (44.37)			
Tumor diameter (cm)	4.51±1.27			
Tumor location				
Frontal lobe	41 (28.87)			
Temporal lobe	58 (40.85)			
Parietal lobe	30 (21.13)			
Others	13 (9.15)			
Distant metastasis				
Yes	39 (27.46)			
No	103 (72.54)			
Family history of glioma			3.133	0.077
Yes	19 (13.38)	5 (4.17)		
No	123 (86.62)	115 (93.33)		
Smoking			0.257	0.612
Yes	54 (38.03)	42 (35.00)		
No	88 (61.97)	78 (65.00)		
Drinking			0.541	0.462
Yes	62 (43.66)	47 (39.17)		
No	80 (56.34)	73 (60.83)		

### 2. Serum lncRNA-ANRIL and SOX9 levels in glioma patients and their diagnostic significance for glioma

**Fig. 1:**
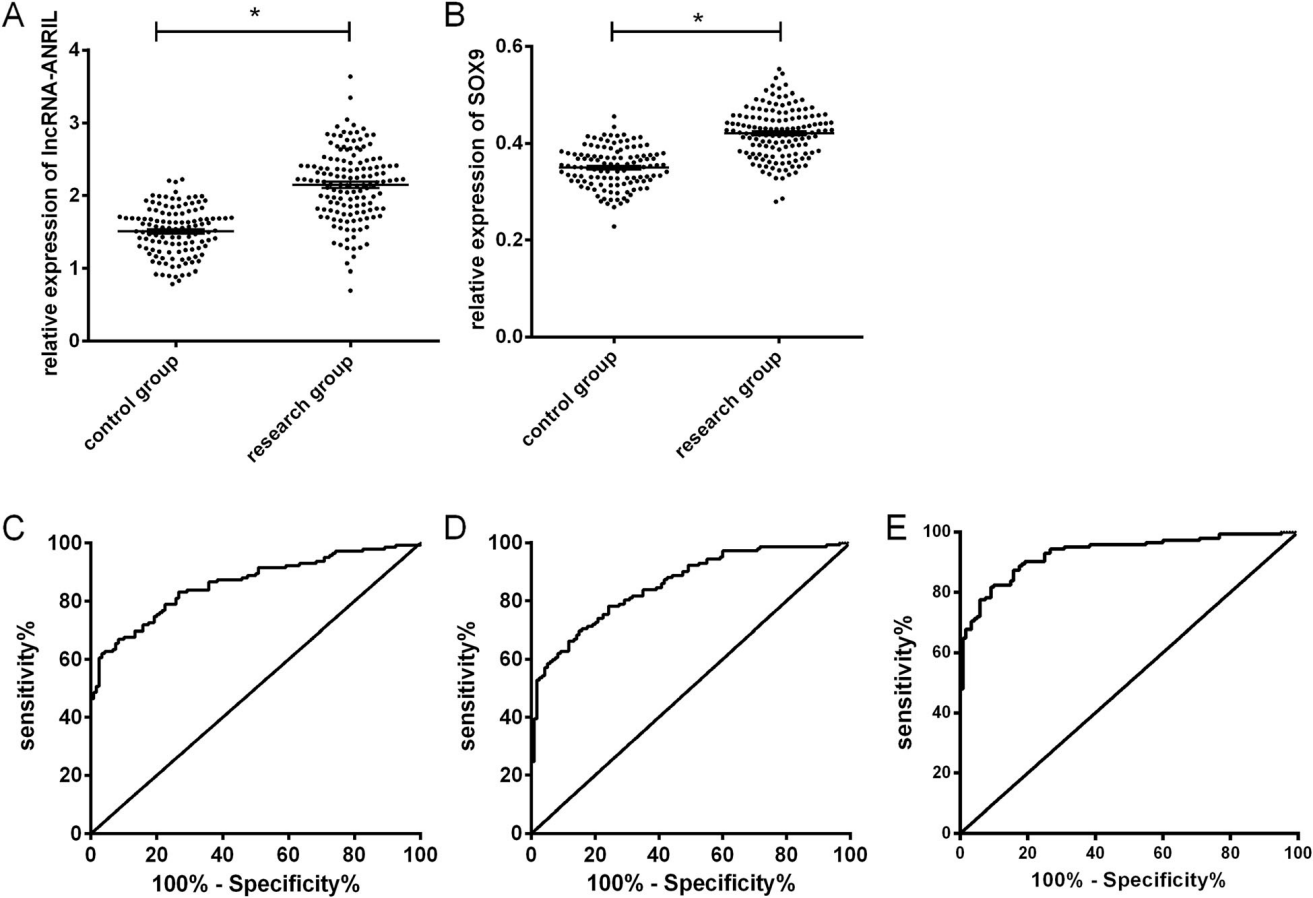
LncRNA-ANRIL and SOX9 expression levels and their diagnostic value. **a** LncRNA-ANRIL levels were statistically higher in RG than in CG. **b** SOX9 levels were notably higher in RG than in CG. **c** Value of lncRNA-ANRIL for the early diagnosis of glioma. **d** Value of SOX9 for the early diagnosis of glioma. **e** Value of lncRNA-ANRIL combined with SOX9 for the early diagnosis of glioma. Note: asterisk indicates *P*<0.01

Serum lncRNA-ANRIL and SOX9 levels were evidently higher in RG than in CG (*P*<0.01) (Fig. 1a, b). The area under the ROC curve (AUC) for glioma diagnosis was 0.860 for lncRNA-ANRIL (Fig. 1c) and 0.857 for SOX9 (Fig. 1d). In the combined detection of the two, RG was used as the independent variable, and a binary logistic analysis was performed to obtain the combined detection model: log (P) = 17.377 + (−3.505) × lncRNA-ANRIL + (−29.307) × SOX9. When the AUC of this model was 0.930, the sensitivity was 81.62% and the specificity was 90.83% for diagnosing gliomas (Fig. 1e, Table 3).

**Table 3:** Value of lncRNA-ANRIL single detection, SOX9 single detection, and the combined detection for the early diagnosis of glioma

	lncRNA-ANRIL	SOX9	Combined detection
Cut-off	2.018	0.395	0.388
Sensitivity (%)	61.97	69.72	81.62
Specificity (%)	96.67	85.00	90.83
AUC	0.860	0.857	0.930
95%CI	91.69–99.08%	77.33–90.86%	84.19–95.33%
Std. error	0.023	0.022	0.016
*P*	<0.001	<0.001	<0.001

### 3. The relationship between lncRNA-ANRIL and SOX9 expression levels and the clinicopathology in patients with glioma

LncRNA-ANRIL and SOX9 were closely related to the increase in tumor grade, tumor diameter, distant metastasis, and family history of glioma (*P*<0.01) Table 4.

**Table 4:** The relationship between lncRNA-ANRIL and SOX9 expression levels and the clinicopathology in patients with glioma

Factors	*n*	Relative expression of lncRNA-ANRIL	*t*	*P*	Relative expression of SOX9	*t*	*P*
Tumor grade			8.650	<0.001		6.621	<0.001
Grade I to II	79	2.47±0.35			0.39±0.04		
Grade III to IV	63	2.02±0.27			0.44±0.05		
Tumor diameter(cm)			3.602	<0.001		6.638	<0.001
≥4.51	77	2.29±0.31			0.45±0.04		
<4.51	65	2.11±0.28			0.41±0.03		
Distant metastasis			5.351	<0.001		5.027	<0.001
Yes	39	2.38±0.27			0.43±0.06		
No	103	2.13±0.24			0.38±0.05		
Family history of glioma			2.166	0.032		2.113	0.036
Yes	19	2.26±0.37			0.36±0.05		
No	123	2.09±0.31			0.40±0.08		

### 4. Prognosis of patients

**Fig. 2:**
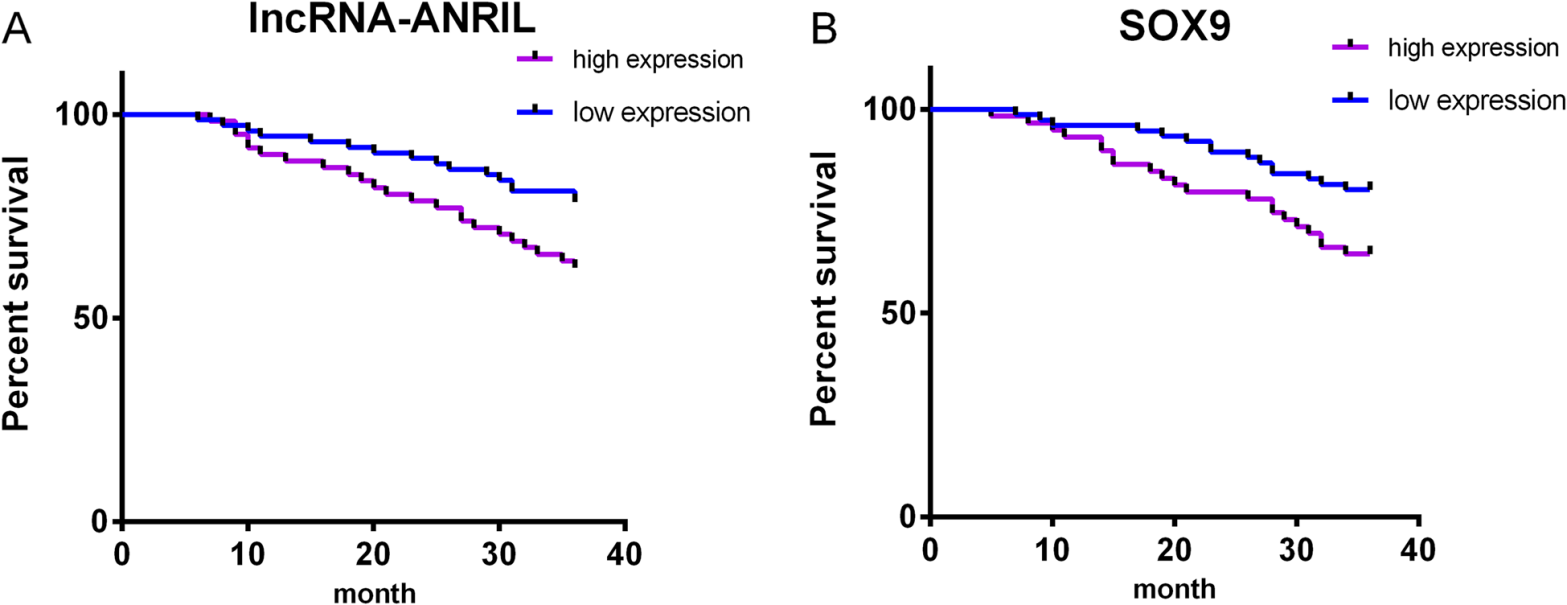
Risk factors affecting the prognosis of glioma patients. **a** The relationship between lncRNA-ANRIL and the prognosis of patients. **b** The relationship between SOX9 and the prognosis of patients

Patients were divided into the high-lncRNA-ANRIL group (≥2.16, 61 cases) and the low-lncRNA-ANRIL group (<2.16, 74 cases) by the median expression of lncRNA-ANRIL, or into the high SOX9 group (≥0.42, 59 cases) and the low SOX9 group (<0.42, 76 cases) by the median expression of SOX9. By May 2019, 135 patients in RG (95.07%) have been successfully followed up by telephone, hospital review, and home visits. The 1-year, 2-year, and 3-year survival rates were 90.16%, 78.69%, and 63.93% in the high-lncRNA-ANRIL group, markedly lower than those in the low-lncRNA-ANRIL group (94.59%, 89.19%, and 81.08%, *P*=0.025) (Fig. 2a). The high SOX9 group had distinctly lower 1-year, 2-year, and 3-year survival rates than the low SOX9 group (93.22%, 79.66%, and 64.41% vs. 96.05%, 89.47%, and 80.26%; *P*=0.039) (Fig. 2b).

### 5. LncRNA-ANRIL expression in glioma cells and its influence on cell multiplication, invasion, and apoptosis

**Fig. 3:**
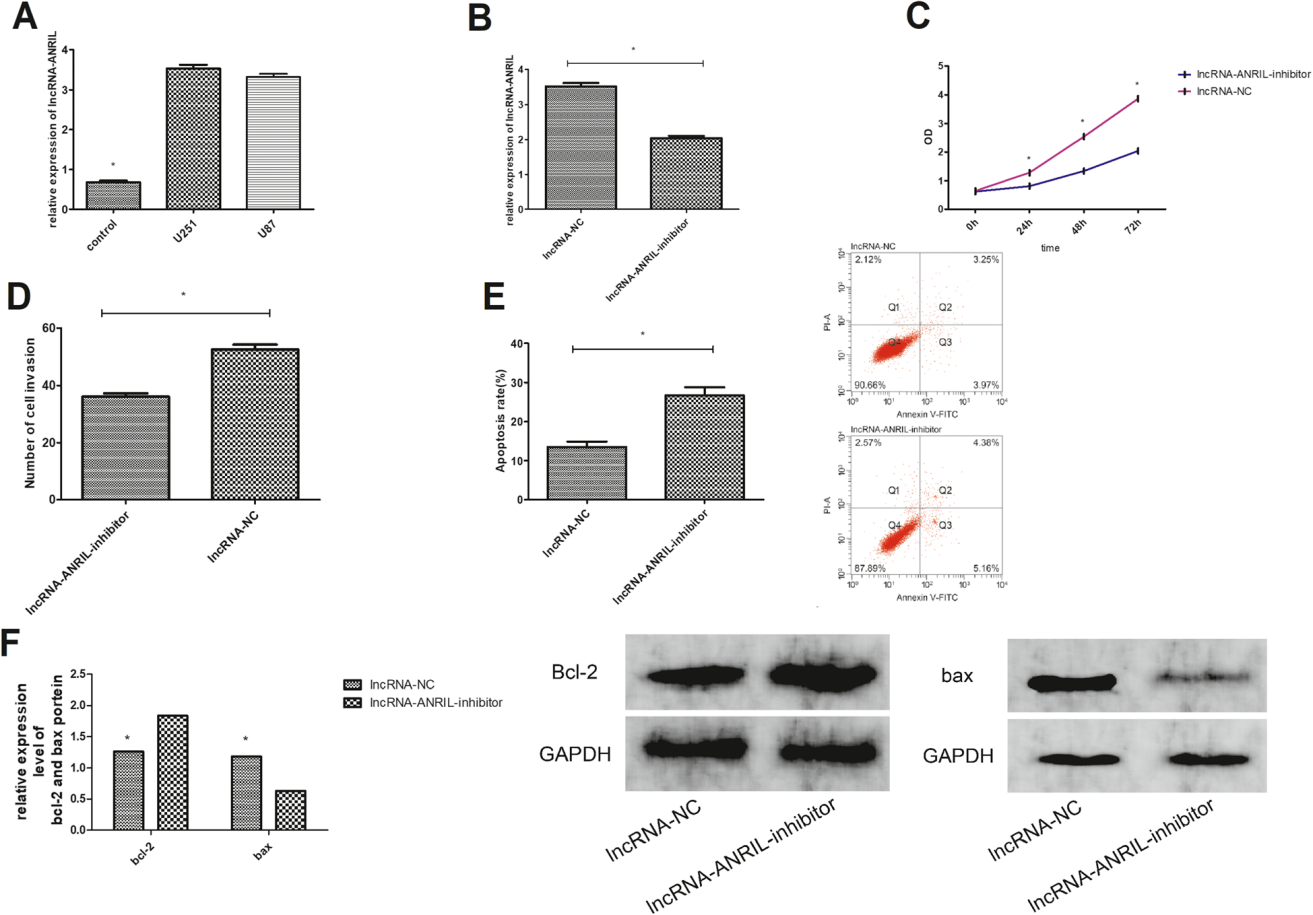
LncRNA-ANRIL expression in glioma cells and its effect on cell multiplication, invasion, and apoptosis. **a** LncRNA-ANRIL showed high expression in glioma cells. **b** LncRNA-ANRIL was markedly under-expressed in cells transfected with lncRNA-ANRIL-inhibitor than in cells transfected with lncRNA-NC. **c** Comparison of cell proliferation. **d** Comparison of cell invasion. **e** Comparison of cell apoptosis and flow cytometry–based cell counting. **f** Expression levels of bcl-2 and bax proteins after transfection and the WB imaging. Note: asterisk indicates *P*<0.01.

LncRNA-ANRIL was markedly higher in U251 and U87 cells than in normal HA (*P*<0.01) (Fig. 3a). After transfection, lncRNA-ANRIL expression was markedly lower in U251 cells transfected with lncRNA-ANRIL-inhibitor than in U251 cells transfected with lncRNA-NC (*P*<0.01) (Fig. 3b). Cells transfected with lncRNA-ANRIL-inhibitor had markedly lower multiplication and invasion and markedly higher apoptosis compared with those transfected with lncRNA-NC (*P*<0.01) (Fig. 3c–e). WB assay revealed that cells transfected with lncRNA-ANRIL-inhibitor had markedly elevated bcl-2 protein expression and reduced bax protein expression than those transfected with lncRNA-NC (*P*<0.01) (Fig. 3f).

### 6. SOX9 expression in glioma cells and its influence on cell multiplication, invasion, and apoptosis

**Fig. 4:**
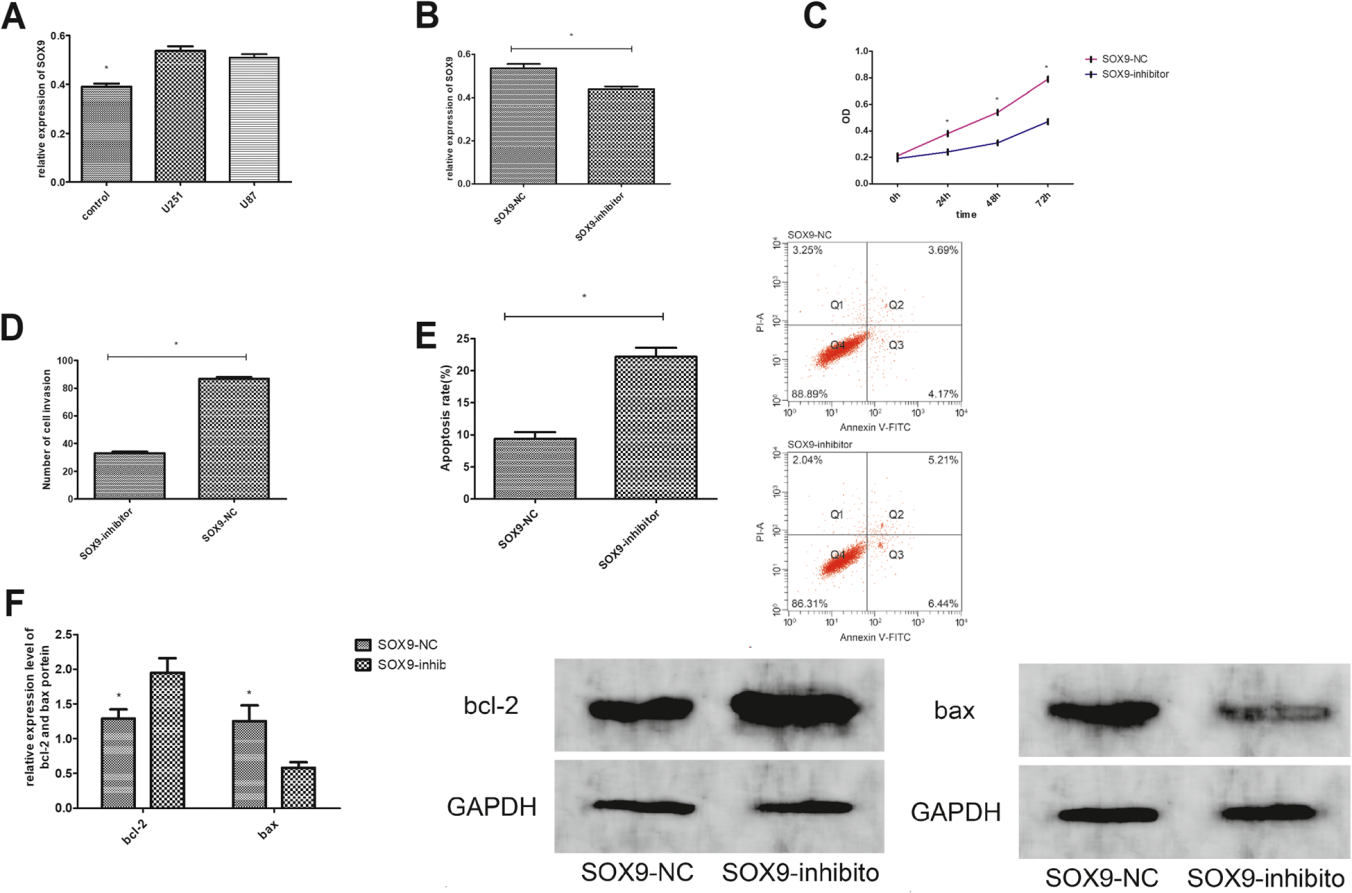
SOX9 expression in glioma cells and its influence on cell multiplication, invasion, and apoptosis. **a** SOX9 presented high expression in glioma cells. **b** SOX9 was markedly under-expressed in cells transfected with SOX9-inhibitor than in cells transfected with SOX9-NC. **c** Comparison of cell proliferation. **d** Comparison of cell invasion. **e** Comparison of cell apoptosis and flow cytometry–based cell counting. **f** Expression levels of bcl-2 and bax proteins after transfection and the WB imaging. Note: asterisk indicates *P*<0.01

The expression of SOX9 was markedly higher in U251 and U87 cells than in normal HA (*P*<0.01) (Fig. 4a). After transfection, SOX9 expression was markedly lower in U251 cells transfected withSOX9-inhibitor than in those transfected with SOX9-NC (*P*<0.01) (Fig. 4b). Cells transfected with SOX9-inhibitor had markedly reduced multiplication and invasion and markedly enhanced apoptosis compared with those transfected with SOX9-NC (*P*<0.01) (Fig. 4c–e). WB assay revealed that cells transfected with SOX9-inhibitor had markedly higher bcl-2 protein expression and lower bax protein expression than cells transfected with SOX9-NC (*P*<0.01) (Fig. 4f).

## Discussion

Glioma is a common malignant brain tumor originating from the neuroectoderm, mainly distributed in the central and peripheral nervous systems of the human body, being the main reason for deaths from brain cancer [22]. It is featured with a high recurrence rate, a poor prognosis, and a yearly increase in its morbidity [23]. Glioma causes no marked discomfort in the early stage until its obvious clinical symptoms occur, accompanied by neurological deficits. Patients are already with grade III to IV malignant glioma tumors in infiltrative growth at the time of diagnosis, showing no clear demarcation between the invaded surrounding normal brain tissue and the tumor, which hinders the clinical treatment of glioma [24]. The existing common treatment for glioma is surgical resection followed by chemotherapy and radiotherapy [25, 26]. Despite the advancement in glioma treatment methods, the puzzle of its underlying mechanism restricts the improvement in the overall treatment efficacy, leading to no reduction in its morbidity and mortality [27]. In recent years, a growing number of scholars have been working on the molecular pathogenesis of gliomas and its gene-related therapies [28, 29]. LncRNA is a type of non-coding RNA with over 200 bases. Among them, lncRNA-ANRIL, an antisense RNA located in the chromosome 9p21.3 with 3.8 kb in length, is expressed in various tissues in the human body [30, 31]. LncRNA-ANRIL has been identified as a genetic susceptibility site for a variety of cancers including intracranial aneurysms [32]. SOX9 is a part of the SRY-related gene family, closely related to tumor development and progression [33, 34]. In-depth studies of glioma have revealed a close correlation of bcl-2 and bax with cell apoptosis. Bcl-2 is an apoptosis inhibitor, but bax is an apoptosis inducer [35]; hence, the determination of bcl-2 and bax levels can be used as a crucial judge of the malignancy of gliomas. The present study investigated lncRNA-ANRIL and SOX9 levels in gliomas and their effects on the biological functions of cells, aiming to enlighten new ways of diagnosis and treatment for gliomas in terms of molecular biology.

Here we employed qRT-PCR to determine serum lncRNA-ANRIL and SOX9 levels in glioma patients and healthy adults and detected abnormally upregulated expression of the two in the serum of glioma patients. Sang et al. also found an upregulation of lncRNA-ANRIL and SOX9 expression in gliomas [36, 37], which is consistent with our results and supports our findings. The ROC curve revealed that when the cut-off value was 2.018, the sensitivity of lncRNA-ANRIL for glioma diagnosis was 61.97% and the specificity was 96.67%; the sensitivity and the specificity of SOX9 for glioma diagnosis were 69.72% and 85.00%, respectively, when the cut-off value was 0.395; and when the cut-off value was 0.388, the combined diagnosis had higher sensitivity than the single diagnosis with lncRNA-ANRIL or SOX9. We further analyzed patients’ clinicopathological characteristics and found that high-lncRNA-ANRIL and SOX9 levels were correlated with tumor grade, tumor diameter, distant metastasis, and family history of glioma. The 3-year survival was markedly higher in the high-lncRNA-ANRIL group and high SOX9 group than in the low-lncRNA-ANRIL group and low SOX9 group, suggesting that lncRNA-ANRIL and SOX9 are related to the prognosis of patients with glioma. So we can monitor lncRNA-ANRIL and SOX9 expression to improve the prognosis of patients. The above results conclude that lncRNA-ANRIL and SOX9 are closely related to the development and progression of gliomas. In our research, lncRNA-ANRIL and SOX9 were higher in U251 and U87 cells than in HA, which is consistent with our previous research results. The expression of lncRNA-ANRIL and SOX9 was markedly reduced in U251 cells by gene downregulation. Then we transfected lncRNA-ANRIL-inhibitor, lncRNA-NC, SOX9-inhibitor, and SOX9-NC to U251 cells to observe the changes in the cell biological functions. Cell proliferation and invasion were restricted after the suppression of lncRNA-ANRIL and SOX9 expression, but the cell apoptosis was remarkably stimulated, which is consistent with the results of Dai et al. [38, 39]. Cells transfected with lncRNA-ANRIL-inhibitor and SOX9-inhibitor had higher bcl-2 levels and lower bax levels compared with cells transfected with lncRNA-NC and SOX9-NC, which is consistent with the results of Jia et al. [40]. Such results suggest that glioma cell proliferation and invasion can be controlled by silencing lncRNA-ANRIL and SOX9. However, the underlying mechanisms of the two to affect the molecular biological functions of glioma cells are not clear.

Here we confirmed high expression of lncRNA-ANRIL and SOX9 in glioma and proved that the inhibition of lncRNA-ANRIL and SOX9 can modulate the multiplication, invasion, and apoptosis of glioma cells. This study is subjected to some limitations. For example, the limited experimental conditions hindered us to figure out the specific regulatory network of lncRNA-ANRIL and SOX9 to affect glioma. We will supplement our conclusions with data in follow-up studies.

## Conclusions

In summary, lncRNA-ANRIL and SOX9 levels were higher in glioma patients than in healthy people. The high expression of lncRNA-ANRIL and SOX9 was closely associated with the unfavorable prognosis of patients. The testing of biological behaviors revealed that lncRNA-ANRIL and SOX9 worked as tumor-promoting genes in glioma.

## Data Availability

The datasets used and/or analyzed during the current study are available from the corresponding author on reasonable request.
